# A Pioneer Tool to Reduce Restrictive Practices toward People with Intellectual and Developmental Disabilities

**DOI:** 10.3390/bs14040344

**Published:** 2024-04-19

**Authors:** Victoria Sánchez-Gómez, Miguel Ángel Verdugo, Manuela Crespo, Amalia San Román

**Affiliations:** 1 Institute for Community Inclusion (INICO), University of Salamanca, 37005 Salamanca, Spain; verdugo@usal.es (M.Á.V.); mcrespo@usal.es (M.C.); 2 Department of Personality, Assessment, and Psychological Treatments, University of Salamanca, 37005 Salamanca, Spain; 3 Plena Inclusión, 28020 Madrid, Spain; amaliasanroman@plenainclusion.org

**Keywords:** disability, restrictive practices, restraints, restrictive interventions, organizational transformation, human rights

## Abstract

Reducing restrictive practices toward individuals with intellectual and developmental disabilities is a globally recognized imperative and human rights priority. This paper presents a novel tool called LibRe for assessing and reducing restrictive practices. This tool involved an instrumental multistage design and collaboration between professionals, individuals with disabilities, family members, and experts from different fields. It addresses diverse restrictive practices in five key domains: physical or mechanical, chemical or pharmacological, structural, relational, and practices related to contexts and supports. It addresses practices that are pertinent to the Spanish context and that existing tools have not covered. Embedded as a step within an organizational approach, LibRe fosters organizational transformation and provides resources to achieve outcomes within reduction plans for restrictive practices. In total, 156 teams comprising 585 professionals, 64 people with disabilities, and 44 family members responded to the tool. In terms of evidence for internal structure validity, the oblique five-factor model exhibited an adequate fit through confirmatory factor analysis, along with satisfactory reliability indices, according to ordinal alpha and omega. Users positively appraised the tool’s usefulness and identified its strengths and challenges. Although further research is needed, preliminary evidence frames LibRe as a useful resource for practice and research.

## 1. Introduction

Restrictive practices—also called restrictive measures—such as restraint, restrictive interventions, and seclusion are traditionally and currently administered to people with intellectual and developmental disabilities (IDDs) in institutionalized settings [1,2,3]. These types of practices have been applied especially, but not only, when individuals with IDDs present challenging behavior [1,2,3,4,5,6].

Restrictive practices denote any kind of act, practice, or intervention that limits the rights or freedom of movement of a person with a disability [7,8]. The terms “restraint”, “restrictive interventions”, and “seclusion” are different concepts, but they all correspond to restrictive practices. Restraint is a restrictive measure that prevents or inhibits a person’s freedom of movement through bodily force (i.e., physical restraint), the use of objects on the body (i.e., mechanical restraint), or the use of medication (i.e., chemical restraint). Restrictive interventions are not restraints, but they limit an individual’s movement or freedom and impinge upon people’s rights, such as prevention from leaving a space, loss of privileges, loss of access to personal property, and increased supervision. Further, seclusion is a restrictive measure that involves confining or separating individuals from others by placing them alone in a room or space from which they cannot freely leave [3,6]. Other practices beyond restraint, restrictive intervention, and seclusion have been understood as restrictive practices, such as the use of psychological strategies to coerce, cultural norms, and blanket rules [9,10]. Restrictive practices refer to a wide range of practices that include the environment, dynamics, atmosphere, and routines [11].

The use of restrictive practices is controversial. For example, ethical concerns about using these practices relate to physical risk, psychological harm, and violation of the rights of the person with a disability [3,12,13]. Regarding physical risk, restraint has reportedly threatened the health and safety of individuals with IDDs who are restrained and the individual performing the restraint, such as staff [3,13]. Regarding psychological harm, physical restraint can elicit memories of abuse and trigger trauma, stress, and a sense of abuse in the individuals who are restrained, as well as feelings of guilt and self-condemnation in those who perform the restraint [1,4,13]. In terms of the rights of individuals with IDDs, these restraint practices limit freedom, dignity, and personal choice [3]. Therefore, even though these practices primarily aim to keep individuals away from harm, and they have been considered the safest method of interacting with individuals exhibiting challenging behavior, conflicting evidence has questioned these practices’ safety [1,3,13]. From the perspective of a quality-of-life (QoL) framework [14], reducing and eliminating unnecessary restrictive practices is essential for improving QoL dimensions such as physical and emotional well-being, self-determination, and the rights of people with disabilities.

It has thus been emphasized that these practices should be eliminated or reduced to the minimum, and in cases where they are considered necessary, their use should be highly justified. Restrictive practices may be justified in exceptional circumstances, for instance, when they are used in response to risk situations (to manage an immediate risk and keep everyone safe) [8]. In this regard, has been emphasized that these practices should always be person centered (to best support them and help them), and they should not aim to control behavior or deliver long-term and lasting behavioral change [8]. According to the Mental Capacity Act 2005 [15], considering whether a practice is the least restrictive means of meeting individuals’ needs and aligning with their rights and freedoms is crucial. Therefore, restrictive practices should only be used in an individual’s best interests, they must be the “least restrictive” method, and they should only be used as the last resort after following other preventive strategies [8,16].

For all the above reasons, regulating and minimizing restrictive practices toward people with IDDs in institutionalized settings has become an issue of international relevance and a human rights concern [1,17,18]. This has also been reflected in research focusing on these practices and the need for their reduction [12,19,20,21,22]. This awareness has also been reflected in policies focusing on the regulation of these practices, e.g., [7,15]. In accordance with the Convention on the Rights of Persons with Disabilities [23], the Attorney General’s Office of Spain developed instruction 1/2022 (January 19) to regulate the use of mechanical or pharmacological means of restraint in health and social health centers [24]. This regulation specifically relates to the Convention on the Rights of Persons with Disabilities article 5 (equality and non-discrimination), article 10 (right to life), article 12 (equal recognition before the law), article 15 (protection against mistreatment), article 17 (protection of personal integrity), article 19 (right to independent life), article 22 (respect for privacy), and article 25 (right to health and informed consent). The regulation also mandates that all centers focusing on the care of individuals in situations of dependency must develop plans that aim to reduce the use of restrictive practices. This instruction represents a significant milestone in the regulation of restrictive practices in Spain.

In the international and local contexts, assessing the use of restrictive practices is a key aspect of developing and implementing plans for their reduction [25,26,27]. Assessments in the field have primarily focused on restrictive practices in terms of organizational culture, attitudes, and strategies [28,29,30,31] rather than on their actual use. For example, the Restraint Reduction Network Checklist [30] aims to help organizations by assessing the strategies implemented to reduce and prevent restrictive practice use. This checklist is founded on various criteria and enables the identification of different areas for improvement.

In terms of tools that assess the use of restrictive practices, two works are worth mentioning. The most relevant work is the Restrictive Practices Review Tool developed by the Restraint Reduction Network (RRN) [32]. This tool is an observational guideline with an open-response format (i.e., text) that addresses seclusion and physical, mechanical, chemical, environmental, and psychological restraint. Although the RRN considered other kinds of restrictive practices, such as cultural norms and blanket rules [33], those are not included in this tool. The Lancashire Safeguarding Adults Board (LSAB) [8] developed the second relevant work, which comprises an exhaustive audit tool that allows an observer (e.g., auditor) to analyze different factors, such as whether restrictive practices are used (Yes/No), the frequency of use, number of users restrained in a six-month period, and type of practice applied (i.e., checklist). It also considers several other aspects, such as conditions of the restraint, availability of trained staff, risk assessment, recording and monitoring of the restraint, and several other organizational elements. Other reported assessments of restrictive practice use in existing literature have comprised observational data, such as daily reports outlining restraint use [22], or other data extracted from institution databases [34].

However, the primary tools that assess the use of restrictive practices were developed to meet legal and organizational needs; they were not structured on a psychometric approach. Therefore, developing a standardized tool that concurrently considers professional expertise and necessary reflection, as well as the psychometric approach, could greatly facilitate the reduction and monitoring processes of restrictive practice use within organizations, as well as research in the field.

Developing a tool that quantitatively measures observable indicators of restrictive practice use offers several advantages. First, a quantitative assessment of these practices helps identify needs and support evidence-based decision-making, such as prioritizing efforts for improvement. Further, this assessment would help evaluate progress in plans to reduce restrictive practices by using a reliable measure and valid indicators. It could also help explore the use of restrictive practices across different organizational levels or different organizations under a common measurement framework. Additionally, it would enable comparisons to be made between specific groups or contexts, and thereby identify potential variables that may be related to the use of these practices (e.g., type of service, presence of challenging behavior, comorbidity, use of alternative means of communication). Therefore, this assessment would help in identifying groups that may be more prone to using restrictive practices. Therefore, having this kind of assessment tool would contribute to the continuous improvement of services and organizations.

This paper presents a tool for assessing and reducing the use of restrictive practices toward people with IDDs, specifically in Spain. The aims were (i) to design the tool; (ii) to analyze the tool’s psychometric properties; and (iii) to explore how the users appraise the tool. This tool aims to promote reflections and organizational change to minimize the use of restrictive practices. It is considered pioneering because it is the first tool that has been developed from a psychometric approach to assess the frequency of restrictive practices being used toward individuals with IDDs or at the organizational level. It not only includes seclusion and physical, mechanical, and chemical restraints but also other relevant restrictive practices, such as relational, structural, and contextual practices. Further, this tool is the first designed with the Spanish context in mind, as well as the first tool of its kind available in the Spanish language.

## 2. Materials and Methods

The present study was conducted in Spain as part of an initiative by Plena Inclusión, a non-governmental and nonprofit entity, aimed at reducing the use of restrictive practices within organizations. Two teams collaborated to conduct the entire project, including a professional team from Plena Inclusión, which maintained contact with the organizations and service users, and a research team from INICO, which was experienced in applied disability research. This study was instrumental and nonexperimental because it aimed to develop a new tool and evaluate its psychometric properties [35]. The study’s scope was also associative because relationships between variables (i.e., items) were analyzed [36].

### 2.1. Procedure

This study implemented a multistage instrumental design that followed steps proposed by Muñiz and Fonseca-Pedrero [37] for test development. These steps were clustered into two large phases (see Table 1), of which the first involved the different steps taken to design the tool and the second involved the steps taken for the tool’s implementation and data analysis.

### 2.2. Participants

#### 2.2.1. Design Phase Participants

Table 1 also lists the experts who were consulted during the different steps of the tool’s design phase. The table details the qualifications and profiles of each expert.

#### 2.2.2. Tool Administration Participants

Regarding the participants who were recruited to administer the tool, 156 teams comprising 585 professionals, 64 people with disabilities, and 44 family members responded. In total, 60.9% (n = 95) of the participating teams performed an assessment at the organizational level (i.e., they assessed the use of restrictive practices within an organization), while 39.1% (n = 61) performed an assessment at the personal level (i.e., they assessed the use of restrictive practices in terms of a specific individual). Regarding the type of service or center in which the tool was used, 37.2% (n = 58) were daycare centers, 36.5% (n = 57) were residences, 11.5% (n = 18) were occupational centers, 11.5% (n = 18) were “others”, 1.9% (n = 3) were senior centers, and 1.3% (n = 2) were family homes. The “others” category included special educational centers and supported living services. All teams provided their group consent to participate before responding to the tool, in which they agreed to the confidentiality of the information. Neither names nor personal data were collected.

#### 2.2.3. Participants of the Focus Group

An online focus group was conducted with the aim of exploring the users’ appraisals of the tool and any problems that emerged from the response process. For this focus group, the researchers invited a small number of participants who qualitatively represented the tool’s potential users. Plena Inclusión selected six organizations in Spain, two from Galicia, one from Madrid, one from Castilla La Mancha, one from Navarra, and one from Andalusia. Seven professionals who had previously responded to the tool participated in the focus group. All responded to the tool in teams that comprised professionals, family members, and individuals with disabilities. All participating professionals consented to the focus group being recorded for analysis, with the study committing to maintaining confidentiality in terms of handling information.

### 2.3. Data Analysis

#### 2.3.1. Tool Psychometric Properties

To provide evidence for the tool’s internal structure validity and to test the relationships between the measured variables, specifically regarding “the parameters specified by the relationships proposed at the theoretical level” [38] (p. 34), the researchers performed a confirmatory factor analysis (CFA) and estimated the model fit. Further, this study hypothesized and contrasted a first-order structure comprising five first-order factors that referred to each area of restrictive practices. This hypothesized model was oblique, meaning that it accepted the possibility of associations between areas. Given that this study did not consider a total score derived from the tool, it did not hypothesize that the structure possessed a large common factor. The contrasted models considered the empirical grouping of items that belonged to the same areas.

This study’s analyses were performed using R studio 12.0 [39], and the interpretation of the fit indices considered the following criteria: (i) the ratio between the chi-squared and its degrees of freedom, which was adequate if its value was less than 2 (χ^2^/d.f < 2); (ii) the root mean square error of approximation (RMSEA), with values below 0.08 and 0.06 indicating an acceptable and good fit, respectively; and (iii) the Bentler–Bonnet comparative fit index (CFI) and Tucker–Lewis index (TLI), with values above 0.9 being adequate and above 0.95 being optimal [40]. Factor loadings were significant for values above 0.30 and *p* values < 0.05 [41]. Further, the ordinal alpha and omega were calculated to provide evidence of reliability. Both are indices of internal consistency that are suitable for variables of a discrete and ordinal nature [42]. The ordinal alpha and omega calculations for each factor used the factor loadings obtained from the CFA. According to Prieto and Delgado [43], values above 0.70 are considered satisfactory indices.

#### 2.3.2. Tool Users’ Appraisals

The tool users’ appraisals were explored according to the opinions they expressed during the focus group. The focus group discussion focused on the following thematic axes: general experience with the tool, relevance of the areas and practices addressed in the tool, difficulties encountered during the response process, usefulness of examples provided for each item, clarity and relevance of response options, response time, and overall tool usefulness. A thematic analysis was performed to identify the tool’s strengths and challenges. All the strengths and challenges indicated by the users were identified and reported.

## 3. Results

This study aimed to (i) design a tool for assessing and reducing the use of restrictive practices; (ii) analyze the tool’s preliminary psychometric properties; and (iii) explore how the users appraised the tool. The study’s main results are presented in three sections, in which the first section outlines the characteristics of the designed tool. The second section presents preliminary evidence of the tool’s psychometric properties, while the third section highlights how the users appraised the tool.

### 3.1. Tool Characteristics

#### 3.1.1. General Framework

The LibRe tool was created in response to the need to address the use of restrictive practices, and the imperative to create more respectful and less restrictive environments in institutionalized settings. Libre is a Spanish word that means “free”, and LibRe is an abbreviation of libre de restricciones, which means “free of restrictions”. Before fully describing the developed tool, this paper must clarify the framework in which the tool is situated and the reason why it was developed. This tool is not intended to diagnose, judge, or audit; rather, it was developed to facilitate organizational transformation and subsequently enhance the fulfillment of rights and improve the QoL of people with IDDs.

**Figure 1:**
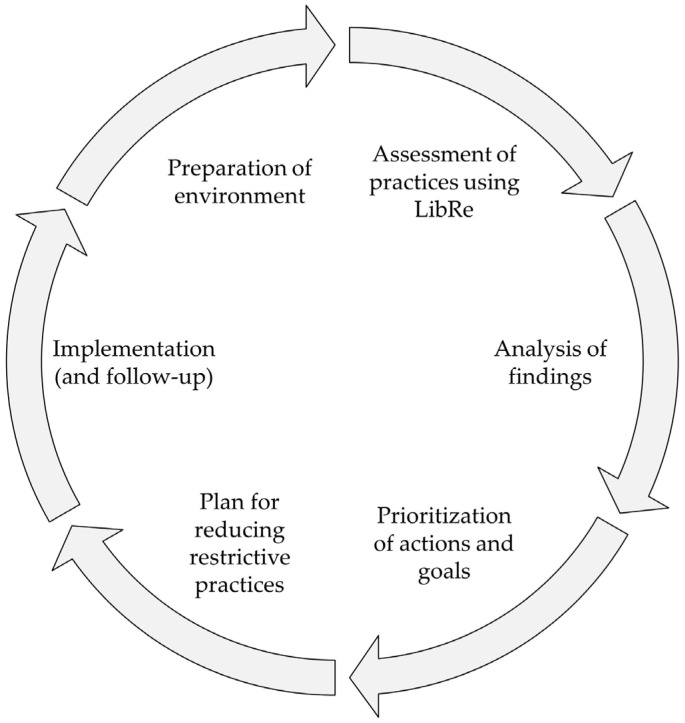
Organizational approach from Plena Inclusión for reducing restrictive practices.

In this proposal, assessing the use of restrictive practices is considered just one step in the process of reducing such practices within organizations. An assessment in itself is useless. It is necessary to allow the implementation of plans to prevent or reduce the frequency of use or restrictiveness [16]. Further, an organizational approach is crucial for successfully reducing restrictive practices [16,25,26,44]. Therefore, the LibRe tool’s intended use is one step of a process to reduce restrictive practices, which involves (i) the preparation of environment, including training and awareness-raising regarding the topic; (ii) a reflexive and systematic assessment using LibRe; (iii) the analysis of the findings; (iv) the prioritization of actions and goals; (v) the elaboration of a plan for reducing or eliminating restrictive practices; and (vi) the implementation and follow-up (see Figure 1). Throughout this process, including all key agents necessary for driving this change is crucial. These agents include direct care professionals or caregivers, as well as individuals with disabilities.

The professional team proposed a set of guidelines for using the results from the tool in a plan, in which they addressed the stages of analysis and prioritization. These guidelines and other materials to support the training of professionals in restrictive practices were included in a floating “more information” tab that remained visible when participants responded to the tool. An English translation for these guidelines is provided in Appendix A, and the original Spanish version is available within the online tool. The LibRe tool also provides a template for a reduction plan together with a detailed results report in .pdf and .csv formats.

#### 3.1.2. Definition of the Variables to Be Measured

Under the broad term of restrictive practices, this study addressed traditional restrictive practices (e.g., restraint, restrictive interventions, seclusion) and other restrictive practices that affect personal freedom and rights [3,9], such as structural practices (e.g., rules), relational practices, and practices related to the context. Additionally, the LibRe tool extends beyond challenging behavior management practices as it includes other situations in which restrictive practices occur (e.g., personal hygiene activities, sexuality, daily routine).

Restrictive practices are thus defined as actions that limit the movement of a part or all of an individual’s body, or their freedom to decide or act independently. These practices can occur in various activities of an individual’s daily life. For example, they can occur during personal hygiene, feeding, leisurely, and sexual activities, as well as during subtle practices that are part of the culture of families, organizations, institutions, and services that provide support.

Table 2 presents conceptual definitions of the areas of restrictive practices that are included in the tool. For the constructs to be measurable, they must be properly operationalized into observable indicators. Therefore, Table 3 outlines examples of indicators for each area.

#### 3.1.3. Tool Specifications

##### Digital Format

The LibRe tool is responded to online through a platform that anyone can use. Users can use the tool at different times and save their progress. The digital format also provides an automatic report to users founded on their responses to items. When users finish responding, they can obtain a results report that details areas and practices exhibiting a higher frequency of use, along with an interpretation of the reported results. Additionally, users can download a .csv document that includes all restrictive practices pertaining to their scores, all the comments they made, and a template to use for their reduction plan that aligns with the guidelines detailed in Appendix A.

##### Response Modalities

The LibRe tool was designed to be answered by work teams (6 to 10 people) that comprise all key agents of daily life practices. For example, this includes directors, professionals, direct care professionals, caregivers, family members, and persons with disabilities themselves. In terms of assessment modalities, the LibRe tool can be used to assess restrictive practices with regard to a specific individual (individual-level assessment), or it can be used to assess restrictive practices at the organizational level. Regardless of the assessment modality, the choice is made before starting the tool.

##### Items Format and Scoring

**Figure 2:**
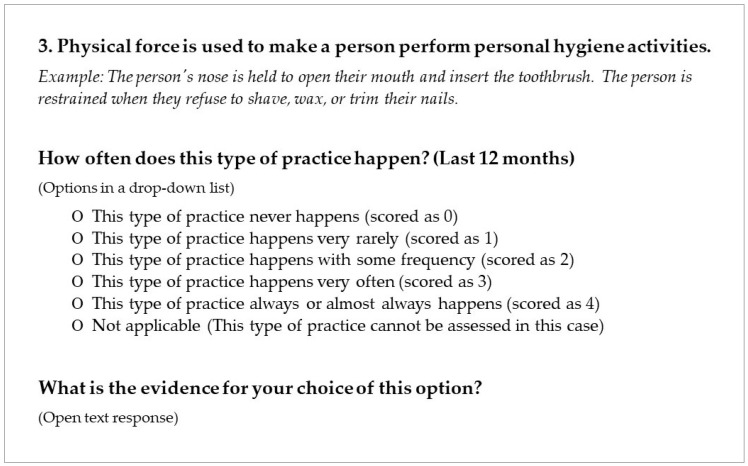
Translated example of an item.

All items have the same response format. They contain a general statement about a restrictive practice and examples of that practice. Respondents are then asked to make a choice by answering, thinking about the last 12 months, “how often does this type of restriction appear?” Respondents are also provided a free-text space to record the reasons for their choice. Figure 2 depicts a translated example of an item that belongs to the “physical or mechanical” area of restrictive practices. A total of 78 items were developed for the pilot version.

Under this response system, a mean is calculated for each area. Therefore, the means per area can be interpreted as follows:

- Score of 0—these restrictions never occur; there are no restrictions regarding these practices; and no actions or attitudes are observed that restrict the individual(s), their activities, actions, decisions, or life in this regard.
- Score between 0 and 1—there are few restrictions regarding these practices; some restrictions of this kind exist; and although they can be evidenced, they are infrequent or occur only under certain circumstances.
- Score between 1 and 2—there is a moderate number of restrictions related to this practice; this type of restriction is common; and it is usual for individuals, their actions, decisions, or activities to be restricted in this regard.
- Score between 2 and 3—there are many restrictions related to these practices; restrictions are very frequent; and it is very common for individuals or their actions, decisions, or activities to be restricted in this regard.
- Score between 3 and 4—there are intense and extensive restrictions regarding these practices; such restrictions are permanent or nearly permanent, and they are part of individuals’ routines of activities, situations, actions, or decisions.

### 3.2. Psychometric Properties of the LibRe Tool

This subsection describes the results pertaining to the preliminary psychometric properties of the LibRe tool, which are founded on data obtained from pilot applications. First, this subsection describes evidence of validity regarding the tool’s internal structure, and then it reports the evidence obtained regarding reliability.

#### 3.2.1. Validity Evidence Based on Internal Structure

The pilot tool included 78 items that addressed a broad set of restrictive practices, which were grouped into five major areas: physical or mechanical (PHM), chemical or pharmacological (CHP), structural (STR), relational (REL), and practices related to contexts and supports (PCSs). The hypothesis referring to the tool’s internal structure was tested using three models (A, B, and C). Furthermore, all CFA contrasts were conducted after purging the database of any responses that contained missing data in any item (n = 117). Table 4 outlines the contrasted models, the number of items considered for each, and the fit indicators obtained.

**Figure 3:**
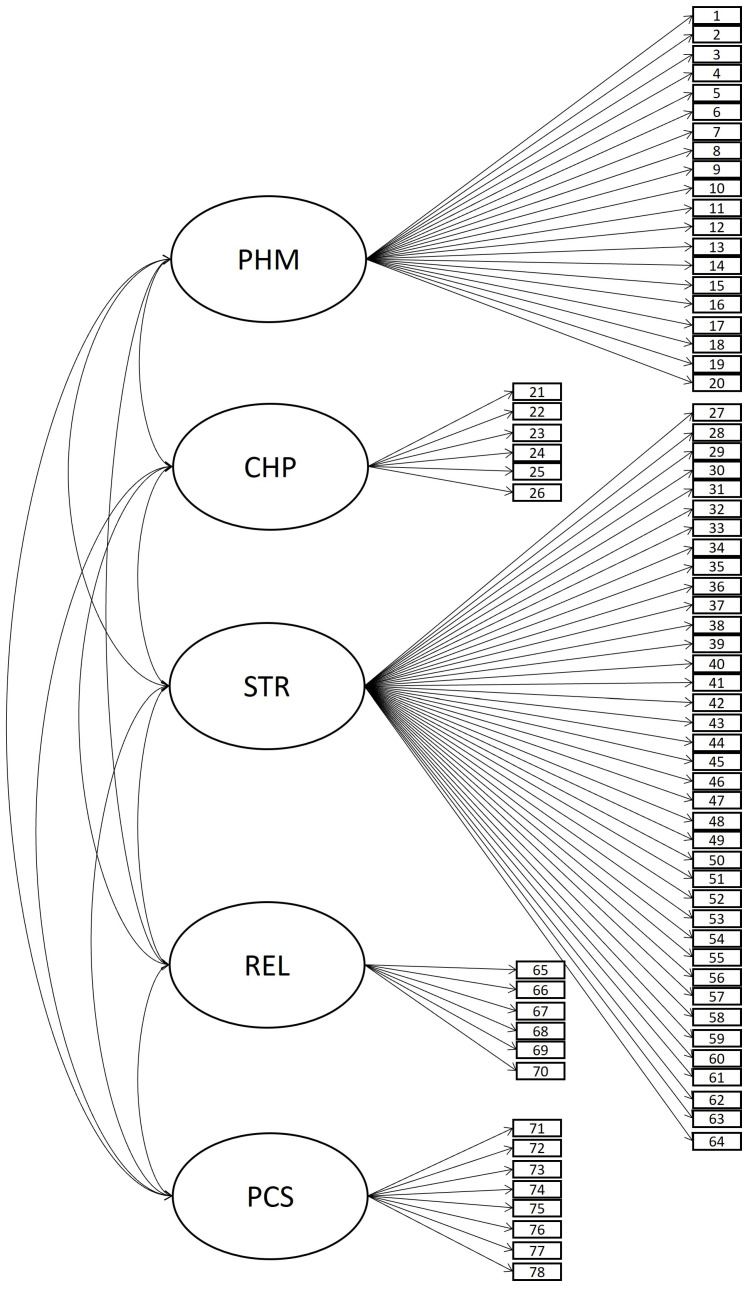
Basal model A, oblique 5-factor model with 78 items.

**Figure 4:**
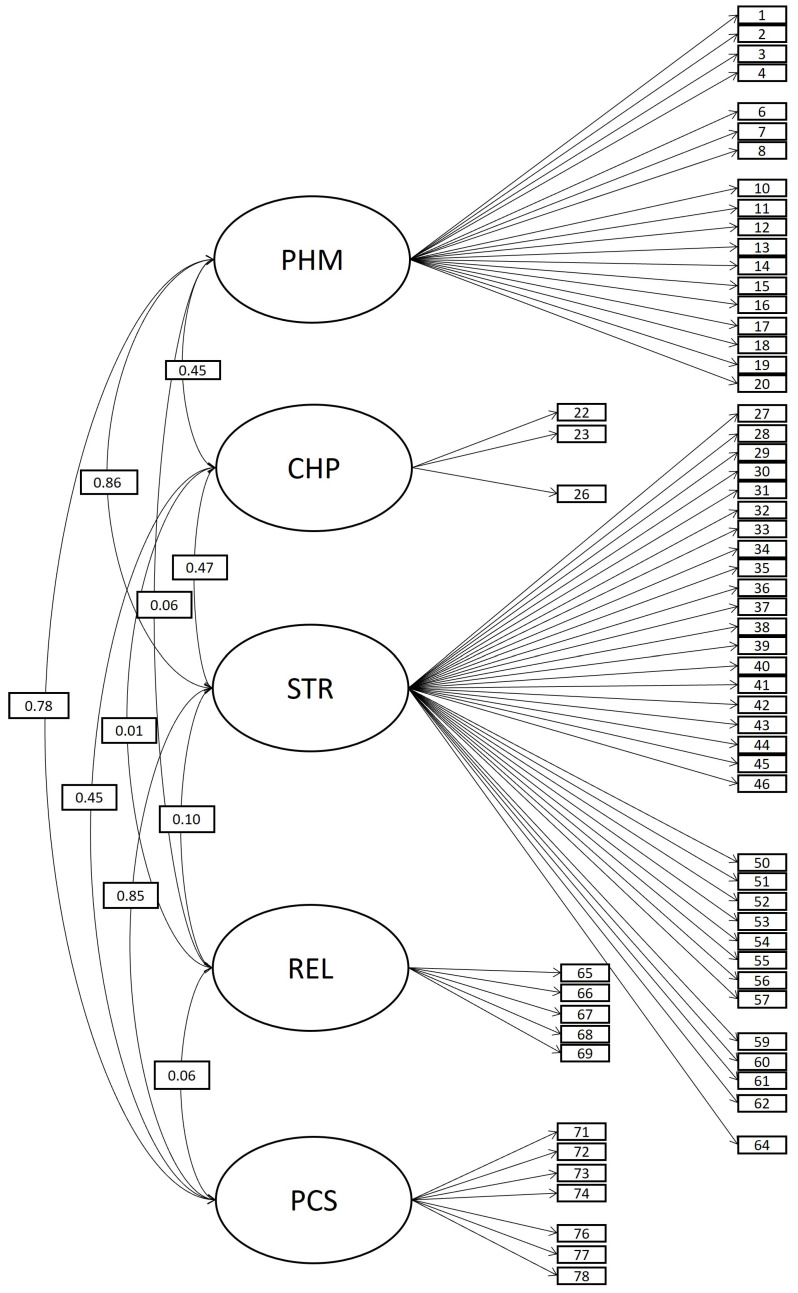
Model B, oblique 5-factor model with 66 items.

For the initial model (model A), the tool comprised five related areas (oblique five-factor model with 78 items; see Figure 3). Although a good fit was obtained for model A according to fit indices (see Table 4), some items exhibited a poor level of performance in terms of standardized factor loadings (under 0.30), R-squared (under 0.10), or variances (equal to or under 0). An alternative model, model B (see Figure 4), considered the same structure as model A, but it was configured with 66 items. The elimination of items that performed poorly caused the varying number of items in the models. Items were discarded because of the value of standardized factor loadings (above 0.30), observed R-squared (above 0.10), and the variance of each item (above 0). The researchers then employed an iterative process, in which the fit indices were re-estimated whenever an item was removed. This sequential process continued until a stable solution was achieved, one that ensured not only a good fit but also that all items exhibited loadings above 0.3, R-squared values above 0.10, and positive variance. Ultimately, this study preliminarily removed the following 12 items in order: 70 (REL), 75 (PCS), 24 (CHP), 5 (PHM), 25 (CHP), it49 (STR), 21 (CHP), 47 (STR), 58 (STR), 63 (STR), 9 (PHM), and 48 (STR). Therefore, these items did not appear in model B (see Figure 4). Due to a low level of correlation between REL and other areas observed in model B, model C considered the same composition as model B (oblique 5F model with 66 items) but included the restriction of null correlation for factor REL. However, model C did not gain much fit. In this way, model B was selected as the most suitable model. The factorial loadings in model B are presented in Table A4. Table A5 presents an abbreviation of the content addressed by each item, as well as the mean, standard deviation, and the minimum and maximum observed for each item.

#### 3.2.2. Evidence of Reliability

The ordinal alpha and omega were calculated for each dimension using the factor loadings of the 66 items in model B. As shown in Table 5, the internal consistency indices were excellent for all areas (above 0.70), except for CHP. This lower level of performance is attributed to the lack of variability observed in responses related to this area (i.e., most participants reported a low frequency for this type of practice). To address this issue and improve model fit during CFA, three items from this area were preliminarily removed. Consequently, only three items remained for this area in model B. Furthermore, the internal consistency indices were highly sensitive to the number of items, which could have been affected by the reduction. This paper’s discussion section delves into potential reasons for the lack of variability and explores implications and future directions related to this area.

### 3.3. Tool User Appraisals

The main appraisals provided by LibRe tool users during the focus group are reported on two axes: strengths and challenges. Because this study’s focus group was conducted in Spanish, some translated quotations from the users are included in the following subsections. The quotes included here are the most representative of all the ideas that emerged in the discussions regarding strengths and challenges. 

#### 3.3.1. Strengths

Overall, users highly valued the tool and emphasized two main reasons. One was that the tool provided a necessary opportunity for reflecting on practice. For example, users stated, “It has allowed us to create a forum to think and pause. It is very important because of the dynamics of the centers, in which we go day after day without stopping. Creating this forum allows us to conclude things and say, ‘well, this is not right,’ and we become aware. I think this is also one of the objectives of this tool”. Other users mentioned that it “is a very useful tool for reflection”, and that “the purpose of the tool is not only to evaluate but also to reflect on the use of these restrictive measures, which is what seems to us to be even more important”. The second reason users emphasized was that the tool provides extensive information. For example, they stated that “it has been a very good experience for us, and it has given us a lot of information”, and that “the tool has been so enriching because of all that we have shared in answering it”. Users further highlighted that the tool was useful for helping families reflect on their own practices. For example, one user stated, “I also liked the fact that you include examples of home practices … I think it is very positive, very useful for the reflection also of families, which are environments that sometimes, due to overprotection, are subject to many restrictions”.

Regarding the tool’s format, the users highlighted the usefulness of the examples, the response option “not applicable”, and the time delimitation (12 months). Regarding the usefulness of examples, users indicated that the examples were key when they reflected on each item or practice. For example, they said, “The examples, which have been key, have helped us a lot”, “examples help a lot”, and “we found the explanations very clear, especially the examples, because the examples did help us situate ourselves”. Some users believed that adding more examples of different settings was needed, but they also highlighted that the existing examples helped them think of more examples. For example, one user noted that “sometimes in some items, we tried to adapt the example to our situation. I mean, okay, we had this example, and that helped us come up with other examples that we adapted to our specific service”.

The response option “not applicable” was highlighted for its usefulness in cases involving a practice that could not be evaluated in a certain environment. One user stated, “In our case, since we are an educational center, it helped us a lot that the answer option ‘not applicable’ appeared.” For the 12-month time delimitation, users appreciated that the items asked them to think about a specific time range. For example, users expressed that “a time delimitation was important and helpful because otherwise, well, you can ramble a lot” and “I consider it necessary that there be a time limitation”.

Regarding the tool’s usefulness in terms of developing future reduction plans, users emphasized two aspects: the usefulness of the qualitative observations for subsequent analysis and the information provided in the floating “more information” tab. The users appreciated that the qualitative observations they made when answering the tool would later appear in the final report. For example, one user expressed, “We find it very useful that the observation that is written at the time of completing the tool appears in the final report below the restriction because it helps us refocus on the restriction that we have identified in our day-to-day work”. After the focus group was conducted, the researchers responded to this feedback and adjusted the tool to incorporate the function of downloadable results (.csv) including each user’s observations.

Regarding the information provided in the floating “more information” tab, users valued this feature because it allowed them to find relevant information about the tool and topic. One user described this as “In our case, it did help us to focus in order to anticipate what we were going to find … in the first session, we used several times the additional information tab that appears on the right-hand side of the platform”. Users also valued the “more information” tab because it allowed them to extend beyond evaluation and achieve real change. As one user stated, “I really liked the ‘more information’ tab because the tool focuses a lot on what not to do or restrictions, but in that little tab, you can find recommendations on how to proceed or training that is important for professionals to receive so that the restrictions are reduced in the organizations”.

#### 3.3.2. Challenges

The main challenge that users identified was the time investment required to respond to the tool, given its length and the broad range of restrictive practices it addresses. As one user described, “It seemed long to us, but we still did it in one go, so I think that is probably not the best way to do it. So, of course it was long, but we had to organize it that way because it took us a while”. However, the users also mentioned that the invested time was worth it, such as this user: “It took us approximately two hours to complete it, maybe a little bit more. And then, well, I mean, it’s long, it is, but it’s certainly worth it too”. Additionally, the users believed that this amount of time was necessary for addressing the tool properly, and they recommend addressing it in different instances to ensure accuracy. For example, one user mentioned that “it has a certain complexity, although I believe that it is not so complex. I think that here, the time factor is important, and maybe you can include some recommendation in that sense”.

Other challenges related to the difficulty of truly including individuals with IDDs in the response process. Users mentioned that the items should be presented ideally in an easy-to-read format to enhance the participation of people with IDDs in the response process: “It is also true that it is not in easy reading, which also makes it difficult. So, we have had to be there, for example, using other words to explain”. However, users also acknowledged that the examples critically helped include these people in the response process. Considering these comments, the current version of the LibRe tool already includes a document of recommendations and strategies to promote the participation of people with IDDs in the response process.

## 4. Discussion

This study presented a novel tool for assessing and reducing the use of restrictive practices on individuals with IDDs. To our knowledge, LibRe represents the first attempt to develop this kind of tool from a psychometric approach, and it is the first Spanish tool of its kind in the field. This study was conducted from a psychological, rights-based perspective, and it was framed within the socio-ecological model of disability. This perspective can be considered a strength, given that most existing research on this topic has primarily focused on medical disciplines, such as nursing. Furthermore, authors have underscored the importance of involving other disciplines in addressing restrictive practices [45].

The use of restrictive practices on individuals in situations of dependency, such as those with disabilities, older adults, or individuals with mental or degenerative illnesses, is a timely and increasingly pertinent topic, e.g., [45,46]. An increasing evidence base for this topic indicates that restrictive practices negatively affect the well-being of individuals who receive the restrictions, as well as the well-being of the staff who implement them [1,13,45]. However, reducing the use of these practices is challenging for organizations. This is because they are primarily practiced as a measure to protect a person or their environment from harm. Therefore, the reduction of restrictive practices must be addressed from an organizational approach that must include a human rights perspective, specialized training, and awareness about these practices [16,25,26,44]. It should not be addressed from a punitive or audit approach to those who implement the restrictions. To drive this change, the process should include all key individuals involved with daily practices, such as direct care professionals or caregivers. This study’s assessment proposal is framed as a necessary step for achieving organizational transformation, and it considers a preliminary step of preparing the environment and subsequent phases of implementing evaluation results to obtain practice change.

Another key aspect of the LibRe tool is that it originated from and was constructed for organizations. To ensure its utility for both practice and research purposes, the tool’s development was founded on a psychometric approach, in alignment with recent literature on psychometrics [37]. This balance ensures the spirit and utility of LibRe. Further, recent literature has emphasized the importance of conducting research collaboratively with organizations and service users [45]. LibRe could be understood as a collaborative tool because it was developed and nurtured from both practice and research expertise. This tool was thus collaboratively developed by organizations and service users such as professionals, families, and individuals with disabilities—those who would use the tool in practice—and by the research sphere within the field of disability and measurement.

As for the conclusions in terms of user appraisals, LibRe was highlighted as a rich tool that provides substantial information. In addition to LibRe’s novelty and collaborative nature, users have highlighted other strengths, such as the tool’s application format. Its digital format allows automatic outcomes to be produced for users, and it could facilitate their use for analyzing and elaborating reduction plans. This is crucial because merely assessing the use of restrictive practices is meaningless without applying the assessment to reduction plans from an organizational perspective [25,26,44]. Another strength is that the response format is consistent throughout the instrument (i.e., all areas are addressed identically). However, we are not excluding the possibility of developing physical versions of LibRe in the future to make it applicable to more contexts. Further, the unconventional use of examples in the items, which is not a common feature in Likert scales, enhanced the analysis process for respondents.

However, LibRe is not free of limitations. The length of the tool might be a challenge for users, as mentioned by the focus group. Some ideas should be highlighted regarding this. The length of the tool is justified from a content perspective because it covers a wide range of practices. Work teams identified all practices included in the tool during the initial stages of its design, and expert judges from different fields subsequently validated their relevance and pertinence. Additionally, the type of practices included may not be identical with other classifications in the field, but they are consistent, e.g., [10]. Further, although users reported a significant time investment to complete the tool, they concurrently emphasized the richness of the information provided.

Additionally, although the ideal scenario in terms of research is to address a tool in its entirety, it is expected that organizations may only choose areas that are relevant to them. For example, one organization might have made significant progress in reducing physical, mechanical, and chemical practices, but they may have not assessed their practices in structural, relational, and contextual aspects. In this case, they could only apply the last three areas. Future research could develop an abbreviated version of the tool that is founded on the most prevalent practices in a target population.

As for the conclusions in terms of psychometric properties, LibRe has shown adequate preliminary evidence regarding its validity and reliability. For validity, preliminary evidence on its internal structure shows a good fit. An adequate structural fit implies that how the tool is answered reflects the conceptual bases that guided its construction [47]. For reliability, the tool yielded excellent indices of internal consistency for almost all areas (except for CHP). However, this evidence should be regarded as preliminary, which is further detailed below.

This study’s most evident and relevant constraint was the size of the sample. The difficulty of obtaining a larger sample size is explained by the group response format, in which each tool response involves an entire group of key informants. Also, the length of the tool poses challenges when assessing psychometric properties in reduced samples. Further studies with a larger sample size are required to establish more conclusive psychometric properties of the tool. Therefore, although a model with 66 items was preliminarily reported, this study’s tool will retain all initial 78 items until more data are collected.

Another important limitation to mention related to the sample size and nature of some restrictive practices is that certain areas with lower variability or prevalence (i.e., CHP) indicate a lower performance in factorial analysis and reliability because of low variability. Specifically, most teams reported that they did not use these types of practices, especially in occupational centers. Therefore, for a more effective analysis of the tool’s performance in this area, larger samples in services and organizations where this kind of practice is more likely to occur should be targeted (i.e., increasing sampling in residential contexts).

Additionally, a larger sample size will allow for establishing evidence of invariance between different forms of application (i.e., at the organizational or individual level), since there may be relevant differences between two different levels of observation. Further, future analysis could explore the internal structure of the PHM and STR areas, which have more items that address varied content. The present study could not achieve this because of the reduced sample size.

Another factor to consider is that evidence of the use of this tool beyond assessment is essential. This includes understanding the tool’s retrospective efficacy in developing reduction plans and monitoring the reduction of restrictive practices. Since LibRe has only been recently launched, this will only be possible after enough time spent using the tool and implementing reduction plans.

Finally, although the examples provided in the tool were developed primarily for people with IDDs in institutionalized settings, LibRe could be useful for other contexts that have similar characteristics. In fact, some of the people who participated in this study’s pilot application came from different contexts, although to a lesser extent (e.g., occupational centers, senior centers). However, this could prompt a lower variability for the items that cannot be evaluated or are highly implausible to occur in those contexts. These cases entail certain future lines of work. Further research is needed on the empirical utility of LibRe in different contexts or with people without IDDs. Additionally, analyzing the applicability of each practice and the need to include more examples relevant to specific contexts is relevant, as is developing parallel versions of LibRe that better respond to the needs of those settings.

This study contributes to the current literature focusing on addressing the restrictive practices toward people with IDDs and their reduction, e.g., [1,2,3,6,7,9,10,12,16,17,18,21,22,30,32]. All these studies align in their pursuit of upholding human rights and improving the QoL of individuals with IDDs. LibRe represents a significant effort toward addressing restrictive practices in Spain. This tool can potentially drive meaningful change and improve the QoL for individuals with IDDs if embedded within an organizational approach. A tool with these characteristics could enable the systematization of reflexive assessments on the use of restrictive practices in organizations, as well as the monitoring of reduction plans. However, further research with larger samples, quantitative and qualitative data on the follow-up of LibRe’s use, and consideration for the development of alternative or abbreviated versions according to the needs of users in different contexts are needed to fully realize LibRe’s potential.

## Figures and Tables

**Table 1:** Tool development procedure.

Phase	Steps ^1^	Participants and Techniques
1. Design phase	Delimitation of the tool’s general framework	The professional team defined the tool’s general framework according to the tool’s intended use. The research team then refined the general framework.
	2. Definition of the variable to measure	Both professional and research teams developed definitions for the areas of restrictive practices from recent literature on the topic. At the operational level, the indicators of the different practices were identified through working sessions involving a small group of intervention experts in highly institutionalized environments and experts in supporting people with challenging behavior. This group comprised professionals and families from the Todos Somos Todas network, which Plena Inclusión and the professional team coordinated. The process was conducted in 2021, and it identified a series of restrictive practices that were relevant to the Spanish context.
	3. Specification of the tool’s characteristics	The professional team devised the tool’s general characteristics, such as digital format, response modalities, and item format, according to the tool’s intended use. The research team then revised and standardized the tool’s instructions, response format, scoring system, and intended visualization.
	4. Item development	From the pool of restrictive practices that the professional team identified in step 2, the research team edited and constructed a series of items that reflected those practices. The research team also compiled an initial version of the tool to be presented to the judges. The professional team previously reviewed and approved this initial version to confirm that all relevant practices were addressed in the tool.
	5. Expert judges’ evaluation	The judges’ evaluations of the tool were central for providing evidence of content validity, and central for obtaining a general valuation of the tool. The expert judges included nine experts from different fields: (i) three judges from disability organizations (two females, one male); (ii) three judges with expertise in disability research (one female, two males); and (iii) three judges with expertise in psychometrics (two females, one male). The judges evaluated each item according to three criteria: pertinence, in relation to the definitions proposed; relevance, in relation to assessing the restrictive practice included in each item; and clarity, in terms of wording. In this process, the judges eliminated and added items, merged others, and made corrections to the wording. The judges also valued conceptual and operational definitions, as well as the tool’s format (e.g., response options, scoring system) and its usefulness. The research team led the analysis in this step. The professional team supported in the selection of judges.
	6. Pilot test editing and assembly	The research team performed the editing step, and the professional team later approved it. The editing step involved considering the judges’ reviews.
	7. Tool administration	In total, 156 teams comprising 585 professionals, 64 people with disabilities, and 44 family members responded to the tool. The professional team managed this process.
2. Implementation	8. Psychometric property analysis	Plena Inclusión provided the database, while the research team performed the data analysis.
	9. Tool users’ appraisal	A focus group was conducted to explore how the users appraised the tool. Seven professionals from six organizations who had previously responded to the tool participated in the focus group.

^1^ The steps were delimited in consideration of Muñiz and Fonseca-Pedrero’s [37] recommendations.

**Table 2:** Conceptual definitions of areas of restrictive practices included in the LibRe tool.

Area of Restrictive Practices	Definition
1. Physical or mechanical practices	Use of direct physical contact, force, or any physical or mechanical device that deprives individuals of their freedom of movement (of a part or all of their bodies), including physical limitations imposed on access to or permanence in certain spaces. This area includes physical contact, use of physical barriers, and restraint with devices.
2. Chemical or pharmacological practices	Use of drugs or medications that are not justified by a diagnosis of illness that deprive individuals of their freedom of movement (of a part or all of their bodies), or that restrict certain behaviors.
3. Structural practices	Use or application of rules that maintain an institution or service’s established and rigid means of operating. This includes practices related to time and activity management, information management, decision-making, money and resource management, and established rules.
4. Relational practices	Use or application of restrictive forms of relationships that professionals or families establish with individuals.
5. Practices related to contexts and supports	Limitations arising from a lack of accessibility to a context in which the lives of the individuals occur, as well as a lack of the necessary support to participate in that context.

**Table 3:** Examples of indicators for each area of restrictive practices.

Area of Restrictive Practices	Examples of Practices Included
1. Physical or mechanical practices	Physical force is used to make individuals perform hygiene activities or take their medication; dressing of individuals is limited, even if they can do it themselves or with active support; objects are used to control individuals’ movement between specific spaces; individuals are confined or isolated; objects that restrict movement or cause discomfort are placed on individuals’ bodies.
2. Chemical or pharmacological practices	Psychotropic drugs are administered to individuals without medical supervision; psychotropic drugs are frequently administered, even if they are prescribed for rescue medication; individuals are overmedicated; medication is abused in crisis situations; the side effects of medications are ignored.
3. Structural practices	Schedules are imposed for eating or hygiene activities without considering individual needs; decision-making autonomy is limited regarding personal matters such as finances, relationships, and daily routines; blanket rules are imposed.
4. Relational practices	Individuals are required to ask permission to access or remain in daily use spaces (e.g., bedroom, rest area, workspace); individuals are subjected to threats or punishment; individuals are manipulated into making certain decisions.
5. Practices related to contexts and supports	Inadequate support is provided for individuals to communicate their needs and desires; insufficient support is provided for individuals to actively participate in their daily activities; cognitive accessibility is restricted.

**Table 4:** Fit indices for each model contrasted by confirmatory factor analysis.

Model		Items	χ^2^/d.f	RMSEA (90% CI)	CFI	TLI
A	Oblique 5F	78 items	1.265	0.048 (0.043–0.053)	0.96	0.959
B	Oblique 5F	66 items	1.108	0.031 (0.022–0.038)	0.987	0.986
C	Oblique 5F with restrictions	66 items	1.125	0.033 (0.024–0.040)	0.985	0.984

Note: restrictions in model C—all factors are related except for factor REL.

**Table 5:** Internal consistency indices for each area.

Area	Items	Ordinal Alpha	Omega
Physical and mechanical (PHM)	18	0.867	0.868
Chemical or pharmacological (CHP)	3	0.615	0.642
Structural (STR)	33	0.941	0.942
Relational (REL)	5	0.810	0.812
Context and supports (PCS)	7	0.852	0.855

## Data Availability

The data are not publicly available due to the privacy of the participants and collaborating organizations. However, the tool is freely available at https://metodologiaspreventivas.plenainclusion.org/ accesed on 1 February 2024).

## Appendix A. Guidelines for Analyzing LibRe Results for a Plan to Reduce Restrictive Practices

**Table A1:** Step 1. Analysis of identified practices.

Key Questions	Guide Questions and Statements
Is this practice necessary?	Is there a less restrictive alternative? What else could we do? Can we change our usual practice? Can we teach the necessary skills to manage the situation without applying the restriction? Can we modify the supports given to favor the reduction of the restraint?
Does it meet the criteria?	It is necessary to prevent significant harm to the person; it considers the emotional effect of the practice (on the person and on those who apply it); the risks involved in implementing this practice have been assessed; it is proportionate, and the issue is important enough to justify the restriction; it is the least restrictive option, it is not more restrictive than necessary, and there is no alternative; all preventive measures have been exhausted; not to be imposed for longer than necessary; balances the interests of the individual and the other people with whom the individual lives or interacts; occurs in the context of a warm, friendly, peer-to-peer, empathetic, and person-centered approach; the person has an outline that helps them understand the restriction; the support team has agreed on the practice, including the individual(s) with IDDs.
What actions will be taken?	Actions or measures should be taken to reduce or eliminate the restriction arising from the analysis and consensus; if the restrictive practice is not necessary, there is an alternative, or if it does not meet the criteria, then improvement actions should be stated, and operational goals should be defined.

**Table A2:** Step 2. Prioritizing actions and goals.

		Impact ^1^	
		High Impact	Low Impact
**Urgency ^2^**	Urgent	Urgent actions with a high impact (first priority)	Urgent actions with a low impact (third priority)
	Non-urgent	Non-urgent actions with a high impact (second priority)	Non-urgent actions with a low impact (fourth priority)

^1^ Impact: valuation of how the specific action and goal related to it has impacted or could affect the life of the person or group of people. ^2^ Urgency: if a specific action and goal related to it can no longer wait, or if it is something that can wait to be addressed in the future.

**Table A3:** Step 3. Elaborating the plan.

Key Questions	Description
What?	Each of the goals is proposed after prioritization.
How?	For each of the goals, the actions and strategies necessary to achieve them are defined.
Who?	The persons responsible for each of the actions pertaining to the goal are established.
When?	A date is established on which each action will begin to be performed, as well as the action’s follow-up date.
Where?	The contexts in which the action is to be performed are delimited.

## Appendix B

**Table A4:** Factor loadings on model B and R-squared for each item.

	*p*	Standardized Loading	R-Squared
**PHM**			
it1	0.000	0.629	0.396
it2	0.000	0.597	0.356
it3	0.000	0.378	0.143
it4	0.000	0.431	0.186
it6	0.000	0.541	0.293
it7	0.000	0.504	0.254
it8	0.001	0.398	0.158
it10	0.000	0.520	0.270
it11	0.000	0.513	0.263
it12	0.000	0.608	0.369
it13	0.000	0.645	0.416
it14	0.000	0.555	0.308
it15	0.000	0.472	0.223
it16	0.000	0.669	0.448
it17	0.000	0.464	0.215
it18	0.000	0.450	0.203
it19	0.000	0.551	0.303
it20	0.003	0.351	0.123
**CHP**			
it22	0.000	0.466	0.217
it23	0.031	0.492	0.242
it26	0.004	0.849	0.721
**STR**			
it27	0.000	0.786	0.618
it28	0.000	0.825	0.680
it29	0.000	0.430	0.185
it30	0.000	0.342	0.117
it31	0.001	0.372	0.138
it32	0.000	0.762	0.581
it33	0.000	0.452	0.205
it34	0.000	0.454	0.207
it35	0.000	0.659	0.434
it36	0.000	0.570	0.325
it37	0.000	0.683	0.466
it38	0.000	0.809	0.654
it39	0.000	0.769	0.591
it40	0.000	0.676	0.457
it41	0.000	0.758	0.574
it42	0.000	0.442	0.195
it43	0.000	0.530	0.280
it44	0.000	0.646	0.417
it45	0.000	0.472	0.223
it46	0.000	0.675	0.456
it50	0.000	0.397	0.158
it51	0.000	0.373	0.139
it52	0.000	0.506	0.256
it53	0.000	0.668	0.446
it54	0.000	0.601	0.362
it55	0.000	0.452	0.205
it56	0.000	0.591	0.350
it57	0.001	0.389	0.151
it59	0.000	0.658	0.433
it60	0.000	0.473	0.224
it61	0.000	0.411	0.169
it62	0.000	0.587	0.344
it64	0.000	0.587	0.344
**REL**			
it65	0.000	0.677	0.459
it66	0.000	0.718	0.515
it67	0.000	0.776	0.603
it68	0.000	0.544	0.296
it69	0.000	0.681	0.464
**PCS**			
it71	0.000	0.757	0.574
it72	0.000	0.815	0.664
it73	0.000	0.698	0.488
it74	0.000	0.476	0.226
it76	0.000	0.598	0.358
it77	0.000	0.677	0.458
it78	0.000	0.689	0.475

## Appendix C

**Table A5:** Content of each item, mean, standard deviation, minimum and maximum observed.

	Content of the Item *	M	SD	Min.	Max.
**PHM**					
it1	Limited participation in personal hygiene activities.	1.36	1.31	0	4
it2	Dressing restriction despite ability.	1.13	1.20	0	4
it3	Physical force used for hygiene activities.	0.34	0.78	0	4
it4	Participation in eating activities limited.	1.01	1.20	0	4
it6	Physical force applied for medication intake.	0.98	1.37	0	4
it7	WC use physically restricted.	1.21	1.59	0	4
it8	Person moved unnecessarily.	1.12	1.44	0	4
it10	Objects used to control person’s movement.	1.96	1.87	0	4
it11	Person’s access to clothing obstructed.	1.74	1.70	0	4
it12	Objects near person restrict movement within a space.	0.30	0.72	0	4
it13	Building closure inhibits individual’s mobility.	0.91	1.51	0	4
it14	Personal belongings stored in inaccessible locations.	0.61	1.23	0	4
it15	Person confined or isolated from others.	1.11	1.50	0	4
it16	Closure of living/work spaces.	0.54	1.10	0	4
it17	Limited individual’s access to own body.	0.61	1.20	0	4
it18	Objects on person restrict movement or cause discomfort.	0.98	1.37	0	4
it19	Person prevented from undressing independently.	1.21	1.59	0	4
it20	Unnecessary device usage prescribed.	1.12	1.44	0	4
**CHP**					
it22	Person given psychotropics without medical supervision.	0.08	0.36	0	*3*
it23	Overmedication during crises or problematic situations.	0.07	0.31	0	*2*
it26	Medication side effects are disregarded.	0.52	1.03	0	4
**STR**					
it27	Leisure activities restricted due to organizational or family reasons.	2.07	1.55	0	4
it28	Duration of daily activities limited disregarding person’s needs.	1.43	1.56	0	4
it29	Participation in desired educational activities restricted	1.15	1.47	0	4
it30	Schedule imposed for food and beverage access.	2.55	1.73	0	4
it31	Smoking moments restricted despite available permitted areas.	1.05	1.60	0	4
it32	Schedule enforced for personal hygiene.	1.84	1.77	0	4
it33	Limited time allocated for personal hygiene	0.70	1.30	0	4
it34	Diaper changing schedule imposed disregarding person’s needs.	0.37	0.94	0	4
it35	Participation in leisure activity choice restricted.	1.67	1.46	0	4
it36	Person’s involvement in educational activity choice limited.	1.18	1.35	0	4
it37	Participation in daily routine decisions limited.	2.12	1.58	0	4
it38	Community involvement opportunities limited for the person.	1.28	1.55	0	4
it39	Opportunities for satisfactory social interaction limited for the person.	1.04	1.48	0	4
it40	Opportunities for exploring new activities and places limited.	0.88	1.31	0	4
it41	Enjoyment of free time in chosen activities limited.	1.19	1.59	0	4
it42	Person’s privacy breached regarding personal information.	0.84	1.30	0	4
it43	Limited knowledge and decision-making regarding medical treatments.	1.34	1.55	0	4
it44	Person’s decision-making regarding sexual life limited.	1.54	1.63	0	4
it45	Information provided to person about daily activities limited.	0.91	1.21	0	4
it46	Information provided to person about life circumstances limited.	1.32	1.37	0	4
it50	Person’s decision-making regarding diet limited.	1.93	1.67	0	4
it51	Person’s choice of companions and timing limited.	1.40	1.48	0	4
it52	Involvement in support decisions limited for the person.	1.90	1.54	0	4
it53	Person’s money management restricted.	1.81	1.77	0	4
it54	Amount of money person can access is restricted.	1.54	1.67	0	4
it55	Limited access to and use of personal hygiene products.	1.16	1.57	0	4
it56	Access to daily spaces restricted without access keys.	1.93	1.86	0	4
it57	Person’s use of alternative communication devices restricted.	0.78	1.43	0	4
it59	Privacy during showering or using the WC limited.	0.99	1.42	0	4
it60	Privacy for sexual behaviors is restricted for the person.	0.62	1.18	0	4
it61	Person’s freedom to leave spaces restricted.	1.89	1.69	0	4
it62	Contact with family or friends limited for the person.	1.02	1.45	0	4
it64	Involvement in setting space organization rules limited.	2.11	1.69	0	4
**REL**					
it65	Person is infantilized.	2.11	1.69	0	4
it66	Constant permission is demanded from the person.	1.57	1.51	0	4
it67	Permission is demanded for access to daily spaces.	1.77	1.69	0	4
it68	Person is threatened or punished.	1.94	1.71	0	4
it69	Person is persuaded to agree with supporter’s desires.	1.62	1.48	0	4
**PCS**					
it71	Insufficient supports provided for person’s communication needs.	0.90	1.27	0	4
it72	Insufficient supports for person’s active daily participation.	0.76	1.10	0	4
it73	Person offered incomprehensible information, hindering decisions.	0.95	1.25	0	4
it74	Insufficient supports for person’s desired living situation.	1.64	1.67	0	4
it76	Insufficient supports for person’s cognitive accessibility.	0.78	1.11	0	4
it77	Insufficient supports for person’s mobility between locations.	0.70	1.15	0	4
it78	Insufficient supports for person’s meaningful relationships.	1.01	1.31	0	4

* An abbreviation of each item’s content is offered for informational purposes. Due to the length and language of the original items, detailed translations are not provided.
